# Cross-sectional associations between residential environmental exposures and cardiovascular diseases

**DOI:** 10.1186/s12889-015-1788-0

**Published:** 2015-04-30

**Authors:** Antony Chum, Patricia O’Campo

**Affiliations:** Department of Social and Environmental Health Research, London School of Hygiene and Tropical Medicine, 15-17 Tavistock Place, London, WC1H 9SH UK; Centre for Research on Inner City Health, St. Michael’s Hospital, 209 Victoria, 3rd floor, Toronto, ON M5B 1C6 Canada

**Keywords:** Multilevel modeling, Health geography, Urban health, Healthy urban planning, Neighbourhood effects, Environmental determinants of health, Social determinants of health, Built environment and health

## Abstract

**Background:**

Prior research examining neighbourhood effects on cardiovascular diseases (CVDs) has focused on the impact of neighbourhood socio-economic status or a few selected environmental variables. No studies of cardiovascular disease outcomes have investigated a broad range of urban planning related environmental factors. This is the first study to combine multiple neighbourhood influences in an integrated approach to understanding the association between the built and social environment and CVDs. By modeling multiple neighbourhood level social and environmental variables simultaneously, the study improved the estimation of effects by accounting for potential contextual confounders.

**Methods:**

Data were collected using a cross-sectional survey (n = 2411) across 87 census tracts (CT) in Toronto, Canada, and commercial and census data were accessed to characterize the residential environment. Multilevel regressions were used to estimate the associations of neighbourhood factors on the risk of CVD.

**Results:**

Exposure to violent crimes, environmental noise, and proximity to a major road were independently associated with increased odds of CVDs (p < 0.05) in the fully adjusted model. While reduced access to food stores, parks/recreation, and increased access to fast food restaurants were associated with increased odds of CVDs in partially adjusted models (p < 0.05), these associations were fully attenuated after adjusting for BMI and physical activity. Housing disrepair was not associated with CVD risk.

**Conclusions:**

These findings illustrate the importance of measuring and modeling a broad range of neighborhood factors— exposure to violent crimes, environmental noise, and traffic, and access to food stores, fast food, parks/recreation areas— to identify specific stressors in relation to adverse health outcomes. Further research to investigate the temporal order of events is needed to better understand the direction of causation for the observed associations.

## Background

Neighbourhood socioeconomic status (SES) is a well-documented contextual factor for cardiovascular disease (CVD). A comprehensive review found that thirty-two studies (out of thirty-six studies of neighbourhood SES) found increased CVD risk in economically deprived areas in at least one population subgroup (i.e. by gender or ethnicity) after individual-level adjustments [1]. A number of studies have investigated neighbourhood SES, and other census-derived variables— including area-level unemployment [2], population density [3,4], proportion of residents in living in high-rise buildings [5], residential stability [6], and urban/rural status [7] as potential factors for CVD risk. Since the publication of Diez-Roux’s systematic review of the evidence linking neighbourhood environments and CVD outcomes [8], newer studies have gone beyond neighbourhood SES to examine neighbourhood environmental factors including the effect of neighbourhood-based social support on the risk of ischemic heart disease [9], the effect of neighbourhood violent crime on the risk of coronary heart disease [10], the effect of neighbourhood level electoral participation on the risk of coronary heart disease [11], and neighbourhood psychosocial hazards (i.e. violent crimes, abandoned buildings, and signs of incivility) and CVDs [12]. In addition, a separate growing body of literature links CVD outcomes with exposure to physical environmental factors including the impact of residential exposure on traffic and its impact on coronary artery calcification [13], the effect of exposure to ambient particulate and gaseous pollutants on coronary heart disease [14], the joint association of air pollution and noise from road traffic with cardiovascular mortality [15], and proximity to road traffic and coronary heart disease mortality [16]. While the list of social and physical environmental factors studied have grown, no studies of cardiovascular diseases (i.e. myocardial infarction, angina, coronary heart disease, stroke, and congestive heart failure) have simultaneously examined a broad set of social, built, and physical environmental factors including characteristics of land-use, the food environment, housing, crime, traffic, and noise into a single study. By studying a broad range of individual and neighbourhood-level determinants of health simultaneously, this study helps to clarify the relative importance of various environmental determinants of cardiovascular diseases [17].

In addition to studies of cardiovascular outcomes, a number of newer studies have explored a broad range of socio-environmental [18-20] and political/policy-level [21] factors associated with obesity, which is an independent risk factor for CVDs [22]. These studies represent significant advancement of our understanding of the environmental influence on obesity (as well as the spatial composition and built environmental components of obesogenic environments); however, a knowledge gap still exist in terms of the strength of associations between a broad range of neighbourhood characteristics and cardiovascular diseases. With increasing recognition that urban planning initiatives may have an impact on health disparities through modifications to urban infrastructure and built environments [23,24], the present study aims to inform research on healthy urban planning by clarifying the associations between a broad range of neighbourhood-level planning related factors and cardiovascular diseases.

### Neighbourhoods and CVD risk

Our study uses a population health perspective to explore determinants of CVD [25], which aims to explain health outcomes simultaneously at the level of individuals (e.g. diet, physical activity, and other health behaviours) and the broader environment. We built on previous studies that explored environmental predictors of CVD risk factors. Studies have found neighbourhood SES to be independently associated with increased incidence of coronary heart diseases [7], stroke in older adults [26], increased incidence of myocardial infarction [27], increased coronary heart disease case fatality [7], increased risk of clinically significant comorbidities after hospitalization for CVD [28], and increased risk factors for smoking, physical inactivity, obesity, diabetes, and hypertension [29-31]. However, a major limitation of this work is the absence of specific built environmental features [8,32]. Potential neighbourhood factors can be usefully grouped by the mechanism through which they impact CVD risk factors including diet, physical activity, psychosocial stress, and air pollution and noise.

Obesity is an independent risk factor of CVD [33], and can be significantly influenced by diet and physical activity [34]. There is evidence to suggest that the local food environment may have an impact on diet. For example, Black Americans’ fruit and vegetable intake increased by 32% for each additional supermarket in their census tract, and white Americans’ fruit and vegetable intake increased by 11% with the presence of 1 or more supermarkets [35]. Another study found that participants with no supermarkets near their homes were 25-46% less likely to have a healthy diet compared to those with the most stores [36]. A US longitudinal study [37] found that fast food availability is independently associated with consumption, but there was no detectable relationship between food store availability and diet quality. However, a recent Toronto-based study found that the positive impact of grocery stores on fruits and vegetable intake was only detectable in people who spend at least 6.51 hours a day at their residential environment (over and above sleeping hours) [38]; however, analysis using the full sample (including people who are home ≤6.5 hours) found no significant association between residential food environment and diet outcome.

Neighbourhood factors may also influence physical activity levels. In a comprehensive review [39], neighbourhood factors including 1) accessibility to facilities for physical activities, 2) awareness and satisfaction of amenities for activities, and 3) aesthetic qualities of the area were independently associated with increased physical activity for adults. However, the majority of studies reviewed were cross-sectional, and 16 out of the 19 studies relied on self-reported measures of environmental attributes, which may suffer from same-source bias because physically active individuals may view their environments differently than non-active individuals. A number of more recent Canadian studies have also found neighbourhood level factors independently associated with obesity including low neighbourhood-level education and dwelling values [18] and low density land uses and low levels of walkability [19]. On the other hand, a study of the Canadian city of Hamilton found no social and physical environmental characteristic (determined by residents’ open-ended responses of their likes and dislikes) to be significantly associated with being overweight, i.e. BMI ≥ 25 [17].

A review of epidemiological and clinical studies concludes that depression and anxiety can increase the risk of CVD in healthy populations [40]. Thus, neighbourhood stressors that impact depression and anxiety, may indirectly impact CVD risk. Matheson *et al.*’s [41] study suggests that residential instability (measured by indicators such as percentage living alone and percent home ownership) and neighbourhood deprivation are key neighbourhood psychosocial stressors that were associated with depression, after controlling for individual level risks. Galea *et al*. [42] also found that residing in a neighbourhood characterized by poor quality built environment (i.e. housing disrepair) was associated with greater likelihood of depression. However, Mair et al’s [43] review of neighbourhood influences on depression concluded that while structural features such as built environment and socioeconomic composition were significantly associated in a number of studies, social processes such as disorder and violence were more consistently associated with depression. One study investigated the effects of neighbourhood psychosocial factors on CVD risk. Sundquist et al. [10] found that both violent crimes and unemployment rate were positively associated with coronary heart disease after individual level adjustment.

Exposure to pollutants including carbon monoxide, oxides of nitrogen, sulfur dioxide, ozone, lead, and particulate matter can lead to adverse CVD outcomes [44-46]. Since vehicular traffic is an important source for these pollutants [47], residential proximity to traffic is an independent factor for increased cardiovascular and stroke mortality rates [48] and myocardial infarction [49]. While traffic counts are often used as a proxy for air pollution, Vedal [50] raises the concern that noise, a traffic-related exposures, may have an impact on CVDs which should be studied simultaneously. Residential neighbourhood noise and traffic can also significantly reduce the duration of sleep and the increase the risk of sleep problems including sleep onset latency, frequent nocturnal arousals, and premature morning arousals [51]. Short sleep duration and poor sleep quality have been associated with an increased risk for developing stroke [52], and shorter sleep duration increases the risk of obesity and weight gain [53,54], which are risk factors of CVDs. We considered the theory of neighbourhood opportunity structure in the selection of area-level predictors [55], which refers to the distribution of social, economic, service, and built environmental resources required for individual health. The theory considers a broad range of environmental characteristics that engender health at the local scale, and we emphasize those that are most likely related to cardiovascular disease development including physical features (e.g. air quality and traffic), the availability of healthy environments (e.g. areas for leisure and physical activity), services to support daily lives (e.g. availability of healthy food options), and sociocultural features of a neighbourhood (e.g. crime rates). While the majority of previous studies have focused on the impact of neighbourhood deprivation on CVD risk, research examining the association between built environment and CVD risk has generally been limited to a few studies of selected social and land use exposures [10,56] and exposure to air pollution/traffic [44-46]. This is the first study to combine multiple neighbourhood influences guided by opportunity structure theory in an integrated approach to understand the association between the built environment and CVDs. By modeling multiple contextual variables simultaneously (along with neighbourhood SES), the study can improve the estimation of effects by accounting for contextual confounders. Based on the literature reviewed above, our study aims to answer the following research question: are neighbourhood social and built environmental factors (i.e. poor access to food stores, parks & recreation areas, exposure to violent crimes, fast food restaurants, housing disrepair, environmental noise, and proximity to a major road) associated with higher risk of CVDs?

## Methods

**Figure 1:**
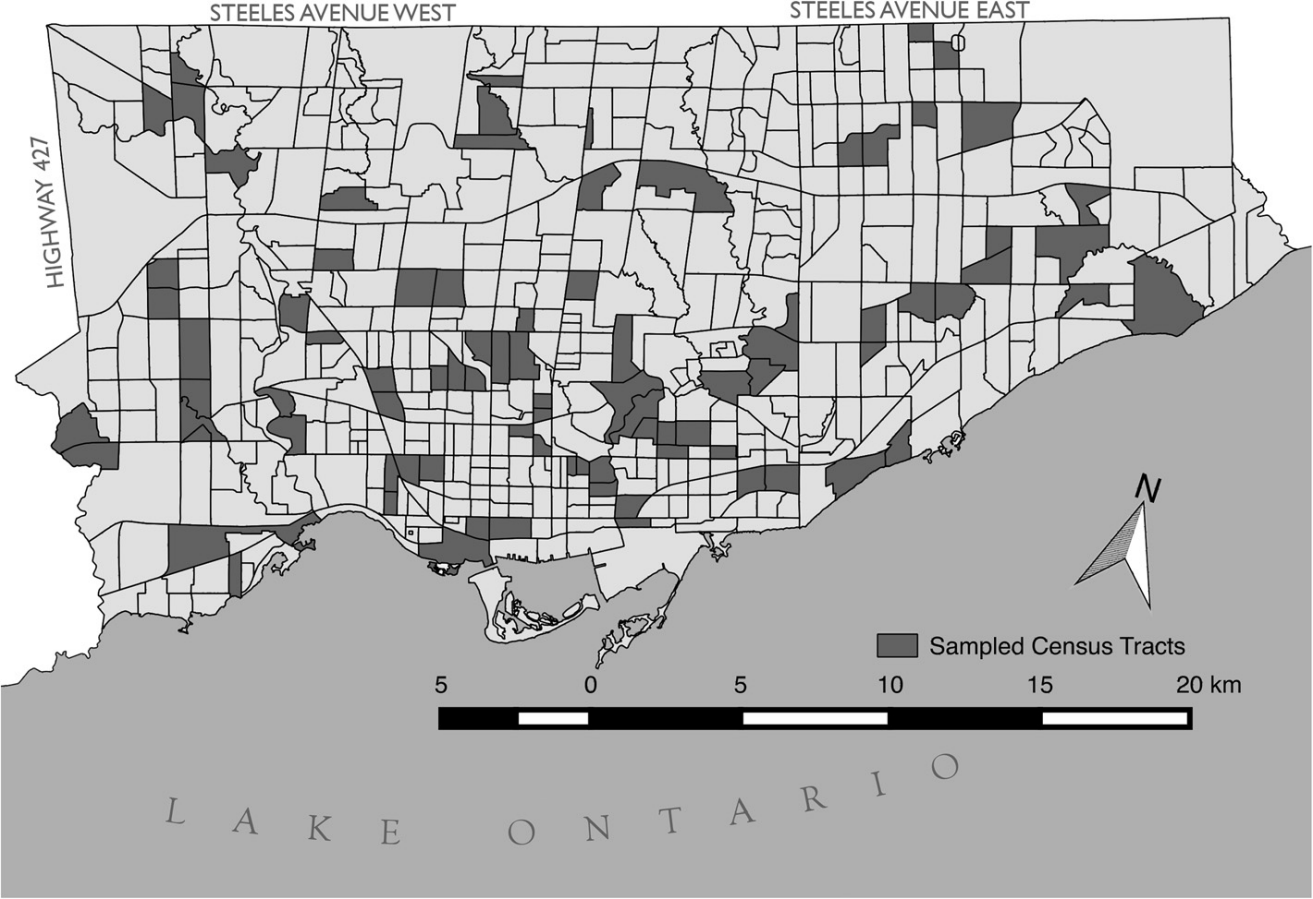
Map of the City of Toronto with the sampled census tracts.

Our study is based on a cross-sectional survey (collected between 2009 and 2011) designed to understand the impact of neighbourhood level determinants on population health. Our study used a three-staged sampling technique: first, 47 out of the total 140 neighbourhood planning areas (NPA) delineated by the City of Toronto were randomly selected; second, 1–2 census tracts (CT) were randomly selected from each NPA, resulting in 87 randomly selected CTs. The selected CTs are displayed in Figure 1 in a map of the City of Toronto. CTs are small geographic units with populations between 2500 and 8000, and are used as a proxy for residential neighbourhoods because they have high internal homogeneity with regards to social and economic conditions [41]. Third, 20–30 individuals were randomly selected within each CT. Individuals were eligible to participate if they were: 1) a resident of the selected household, 2) between 25 and 64 years of age, 3) able to communicate in English, and 4) lived in the neighbourhood for at least 6 months. Our response rate was 72% (n = 2411). Data were obtained from in-person interviews administered by trained interviewers. All participants provided written informed consent at the time of their interview. The Research Ethics Board at St Michael’s hospital (Toronto, Canada) provided ethics approval for this study [57].

To ensure that our data is representative of our target population, sampling weights were created based on 2006 Canadian Census data for the City of Toronto [58]. The data were weighted by the following socio-demographic characteristics: sex, household income, household size, immigrant status, and age. These variables were chosen because descriptive analyses suggest that our sample is either over or under-represented on each of these characteristics.

### Outcomes

The CVD outcomes were self-reported history of physician diagnosis of myocardial infarction (MI), angina, coronary heart disease (CHD), stroke, and congestive heart failure (CHF). Although this survey did not include clinical details, previous studies of the validity and reliability of self-reported conditions have suggested a high level of agreement with medical records for most of the conditions considered here [59,60]. This study examined 2 outcomes with increasing prevalence for the purpose of checking the robustness of the findings: outcome 1 included self-reported MI, and outcome 2 included self-reported MI, angina, CHD, CHF, or stroke.

### Individual level factors

We examined the effects of the following socio-demographic factors: age, household income, gender, visible minority status, and education. We also examined the effects of lifestyle factors including smoking (i.e. non-smoker, current-smoker, or former-smoker), drinking (light use defined as 0–2 days use per week, moderate defined as 3–4 days use per week, and heavy defined as 6–7 days use per week or more than 5 drinks in one day), physical activity (at least 2.5 hours per week [61]), and body mass index (BMI).

### Social and built environmental factors

Data on access to 1) food stores and supermarkets and 2) fast food restaurants were obtained from the City of Toronto Public Health food inspection reports [62]. Addresses were geocoded using GeoPinpoint v. 3.3 (DMTI, Markham, On, Canada), and the data were imported into ArcGIS Editor 9.3 (ESRI, Redlands, CA, USA) for spatial data analysis. To account for food stores that are adjacent to participants but not in the study CTs (i.e. edge effect), we drew on Sadler et al’s technique [63] to construct 750 meters network buffers (approximately 10 minutes walking distance) around each participant to account for foods stores in these areas. The number of food stores within the CT was then summed with these additional edge stores and subsequently normalized by the total area (i.e. CT area plus additional edge areas) to create a density value. The quartiles for supermarkets and food stores per km^2^ and fast food restaurants per km^2^.were derived.

Information on parks and recreational facilities were included in order to consider the impact of built environmental context on physical activity. Land use information from the CanMap geo-database [64] was used create quartiles for the percent of local area used for parks or recreational facilities. Edge effects were accounted for using the technique described above.

Two sources of neighbourhood-level psychosocial stress are examined. First, the *uniform crime reporting database* (Statistics Canada, 2010) provided data to calculate the number of violent crimes, which included incidents of sexual assault, criminal harassment, uttering threats, minor/major assault, and robbery. We created quartiles for violent crimes per capita at the CT level. Neighbourhood violent crime (normalized by the number of inhabitants in that neighbourhood) has been studied in relation to the risk of coronary heart disease [10]. Second, we created quartiles for the percent of housing requiring major repairs across CTs using data from the Canada census 2006 [65]. Similar metrics of housing quality, as measured by self-reported state of housing disrepair, has been examined as an environmental determinant of health as described in a systematic review of the subject [66].

Traffic data obtained from Toronto Transportation Services [67] is used as a proxy for air pollution. Average weekday 24-hour traffic volume was recorded in the geo-database of a street file in ArcGIS, then 100-meter circular buffers were created around each participant’s home location. Using buffer analysis, those who resided within 100-meters of a major road, defined by the City of Toronto as having at least an average of 20,000 vehicles in a 24-hour period, were coded as exposed, otherwise unexposed. This method of categorizing traffic exposure and the use of a 100-meter buffer zone has been used previously to investigate the effect of exposure to traffic on mortality rate advancement periods [48]. With regards to noise, participants were asked whether they agreed with the statement “the noise level where I live often disturbs me” with the answer chosen on a 5-point Likert scale. Lastly, neighbourhood SES is measure by median after-tax household income (in ten-thousands) from the Canada census 2006.

### Statistical analysis

The analysis began by evaluating the bivariate association between individual level and neighbourhood level predictors and self-reported history of outcome 1 (MI only) and outcome 2 (any of MI, angina, CHD, CHF, or stroke) using χ^2^ test for pairs of categorical variables and 1-way ANOVA for comparing continuous and categorical variables. Since the data has a 2-level structure, with many individuals nested in neighbourhoods, multilevel logistic regression is used to account for the lack of spatial independence [68]. We then calculated the intraclass correlation in a null model to analyze how much of the variance in each of the 2 outcome measures can be potentially attributed to the CT level. This is followed by a series of models: model #1 examines the unadjusted association between neighbourhood level SES and the 2 outcomes; model #2 adds to model #1 by including all other neighbourhood level social and built environmental factors; model #3 further builds on model #2 by adjusting for individual level socio-demographic risk factors and health behaviours. Finally, a fourth model is added where we further adjust model #3 for BMI and physical activity. This step-entry method allowed us to understand the effect of adjusting for individual level risks on the neighbourhood level factors. Multilevel logistic regression was performed using SAS 9.3 in the GLIMMIX procedure.

## Results

### Descriptive analysis

Participants were 53% female and 44% self-reported as visible minority, with a mean age of 44 years (Table 1) and a mean after-tax family income of $91,330 (median = $71,000). Those who have CVDs have a mean age of 48 and a mean after-tax family income of $60,512 (median = $45,000). The mean duration of residence in their current neighbourhood was 9.4 years (range = 0.5-65 years, SD = 8.9). Overall, 47 (1.94%) reported a previous history of MI, and 103 (4.27%) reported a previous history of any CVDs (any one of MI, angina, CHD, CHF, or stroke). Bivariate associations between the outcomes and selected socio-demographic characteristics are presented in Table 1.

**Table 1:** **CVD Outcomes and Characteristics of Study Participants**

		**Outcome 1: MI only**		**Outcome 2: Any of MI, angina, CHD, CHF, or stroke**	
	**Entire Cohort No. (% of sample)**	**No. (row %)**	**p-value**	**No. (row %)**	**p-value**
**Participants**	2411 (100)	47 (1.94)		103 (4.27)	
**Age**			0.0354		<.0001
25-35	529 (21.93)	6 (1.11)		6 (1.21)	
36-45	742 (30.79)	9 (1.20)		20 (2.67)	
46-55	714 (29.63)	19 (2.68)		44 (6.16)	
56-65	426 (17.65)	13 (3.00)		33 (7.67)	
**Gender**			<.0001		0.0105
Women	1293 (53.64)	10 (0.75)		43 (3.29)	
Men	1117 (46.36)	37(3.31)		48 (5.40)	
**Visible Minority Status**			0.7446		0.3990
1. Yes	1062 (44.04)	19 (1.83)		41 (3.87)	
2. No	1349 (55.96)	27 (2.02)		62 (4.57)	
**Education**			0.8194		**<.0001**
1. Less than High School	95 (3.95)	1 (1.45)		13 (14.11)	
2. High School Complete	389 (16.15)	6 (1.49)		12 (3.21)	
3. Diploma Complete	686 (28.44)	16 (2.27)		29 (4.17)	
4. Completed Undergrad and above	1241 (51.47)	24 (1.93)		48 (3.89)	

City of Toronto, Canada 2009–2011 (n = 2411).

Note: MI = Myocardial infarction; CHD = coronary heart disease; CHF = congestive heart failure.

For variables with more than 2 categories, p-value is for the χ^2^ test of differences across row in the percentage reporting the outcome. Otherwise p-value is for a 2-tailed test of difference between 2 groups in the proportion reporting each outcome.

### Intraclass correlation (ICC)

We calculated the ICC using a fully unconditional ordered logit model of the dichotomous outcome of MI (outcome 1) and any CVD (outcome 2), which is calculated to decompose the variance in the predictors across individual level versus CT level of the analysis [69]. The estimate of the intercept variance is 0.14 for MI (p < 0.001) and 0.16 for any CVDs (p < 0.001), which means the CT level can explain 4.08% of the variance in MI and 4.53% of the variance in any CVDs. The ICCs indicate that there may be clustering of individuals with similar characteristics at the CT scale, and/or the existence of CT factors that may shape CVD risk, and more generally, the lack of independence for within-group observations.

### Multilevel logistic regressions

Table 2 reports a series of multilevel logistic regressions predicting outcome 1 (MI only) and outcome 2 (any CVDs). Model 1 estimates the bivariate odds of MI (outcome 1) and all CVD (outcome 2) based on neighbourhood income (not shown on table). We find that an increase in neighbourhood income by $10,000 is associated with reduced odds for MI (O.R = 0.9, p < 0.05) and all CVD (O.R. = 0.92, p < 0.05). The variance of the random intercept remains significant for both outcomes: with ICC at 3.25% and 3.7% respectively for outcome 1 and 2. This indicates that there is still unexplained variance at the CT level (p < 0.001) after accounting for neighbourhood income.

**Table 2:** **Multilevel Logistic Regressions of outcome 1 (MI only) and outcome2 (any CVDs)**

	**Model 2**		**Model 3**		**Model 4**	
	**Model 1** ^**‡**^ **+ all neighbourhood factors**		**Model 2 + selected individual factors**		**Model 3 + BMI and physical activity**	
	**MI only: Odds ratio (95% CI)**	**Any CVDs: Odds ratio (95% CI)**	**MI only: Odds ratio (95% CI)**	**Any CVDs: Odds ratio (95% CI)**	**MI only: Odds ratio (95% CI)**	**Any CVDs: Odds ratio (95% CI)**
Total (n = 2411)	**Neighbourhood level factors**					
**Median household income (in $10,000)**	0.92 (0.85-0.98)*	0.96 (0.94-0.98)*	0.94 (0.86-1.02)	0.98 (0.94-1.02)	1.02 (0.89-1.15)	1.16 (0.79-1.53)
**Food stores density**						
Q1 (low) vs. Q4 (high)	1.60 (1.11-2.10)**	1.48 (1.07-1.90)**	1.23 (1.02-1.44)*	1.12 (1.01-1.23)*	1.07 (0.99-1.15)	1.03 (0.92-1.14)
Q2 vs. Q4	1.51 (0.99-2.02)	1.39 (0.95-1.83)	1.24 (0.95-1.53)	1.04 (0.89-1.19)	1.04 (0.90-1.18)	1.02 (0.87-1.17)
Q3 vs. Q4	1.43 (0.92-1.93)	1.18 (0.82-1.54)	1.09 (0.91-1.27)	0.99 (0.80-1.18)	1.01 (0.89-1.13)	0.99 (0.78-1.2)
**Fast food density**						
Q1 (low) vs. Q4 (high)	0.89 (0.81-0.97)*	0.9 (0.83-0.96)*	0.95 (0.91-0.98)*	0.97 (0.95-0.99)*	1.00 (0.94-1.07)	1.01 (0.93-1.09)
Q2 vs. Q4	0.96 (0.94-0.99)*	0.99 (0.90-1.09)	1.00 (0.93-1.07)	1.02 (0.96-1.08)	1.01 (0.84-1.18)	1.07 (0.87-1.27)
Q3 vs. Q4	1.01 (0.8-1.22)	1.01 (0.91-1.11)	1.02 (0.86-1.15)	1.01 (0.94-1.08)	1.03 (0.74-1.32)	1.04 (0.81-1.27)
**Proportion CT Parks and recreational**						
Q1 (low) vs. Q4 (high)	1.19 (1.10-1.31)**	1.17 (0.31-2.03)	1.1 (1.02-1.18)*	0.98 (0.44-1.52)	1.05 (0.80-1.30)	1.03 (0.11-1.95)
Q2 vs. Q4	1.07 (1.01-1.13)*	1.13 (0.21-2.05)	1.02 (0.73-1.31)	0.99 (0.27-1.71)	1.09 (0.63-1.55)	1.15 (0.22-2.08)
Q3 vs. Q4	1.0 (0.89-1.11)	1.03 (0.53-1.53)	1.02 (0.84-1.20)	1.17 (0.46-1.88)	1.06 (0.72-1.40)	1.21 (0.47-1.95)
**Violent crimes per capita**						
Q1 (low) vs. Q4 (high)	0.85 (0.78-0.92)*	0.79 (0.71-0.87)***	0.87 (0.76-0.98)*	0.80 (0.71-0.89)***	0.93 (0.87-0.99)*	0.82 (0.72-0.92)**
Q2 vs. Q4	0.89 (0.82-0.96)*	0.84 (0.73-0.95)**	0.93 (0.85-1.01)	0.88 (0.79-0.97)*	0.94 (0.79-1.09)	0.90 (0.78-1.02)
Q3 vs. Q4	0.91 (0.80-1.02)	0.89 (0.67-1.11)	0.98 (0.87-1.09)	0.95 (0.72-1.18)	1.02 (0.86-1.18)	1.00 (0.74-1.27)
**Proportion of housing requiring major repairs in CT**						
Q1 (low) vs. Q4 (high)	0.79 (0.28-1.30)	0.66 (0.25-1.07)	0.90 (0.61-1.19)	0.97 (0.48-1.46)	0.94 (0.62-1.10)	0.99 (0.51-1.47)
Q2 vs. Q4	0.83 (0.42-1.24)	0.80 (0.45-1.15)	0.92 (0.48-1.36)	1.00 (0.47-1.54)	0.96 (0.51-1.41)	1.03 (0.54-1.52)
Q3 vs. Q4	0.91 (0.6-1.22)	0.74 (0.37-1.11)	0.94 (0.55-1.33)	1.02 (0.46-1.58)	0.98 (0.57-1.39)	1.09 (0.82-1.36)
**Within 100 m of a major road**	4.07 (2.91-7.56)***	2.33 (1.45-3.97)**	3.86 (2.39-6.13)***	2.01 (1.87-2.55)**	3.79 (2.25-5.53)***	1.97 (1.67-2.49)**
**Disturbed by noise at home** ^**1**^						
1. Strongly Agree	5.21 (1.88-15.78)***	3.37 (1.61-6.75)***	4.84 (1.69-13.63)**	3.25 (1.59-6.19)**	4.83 (1.65-13.20)**	3.19 (1.55-6.25)**
2. Agree	1.51 (0.82-4.91)	1.48 (0.84-3.83)	1.41 (0.67-4.42)	1.39 (0.41-3.74)	1.38 (0.55-4.01)	1.23 (0.47-3.66)
3. Neither agree or disagree	1.28 (0.59-3.89)	1.17 (0.60-3.69)	1.25 (0.52-3.72)	1.16 (0.60-3.79)	1.07 (0.52-3.59)	1.01 (0.55-3.60)
4. Disagree	0.96 (0.48-4.56)	1.06 (0.25-1.25)	1.04 (0.19-3.17)	0.79 (0.26-1.94)	0.99 (0.40-4.12)	0.80 (0.33-2.01)
	**Individual level factors**					
**Age (in years)**			1.05 (1.01-1.09)**	1.07 (1.04-1.09)***	1.04 (1.01-1.08)**	1.07 (1.04-1.09)***
**Male**			4.91 (2.51-9.63)***	1.73 (1.15-2.59)**	5.29 (2.60-10.76)***	1.71 (1.14-2.56)**
**Visible Minority**			0.61 (0.30-1.25)	0.86 (0.55-1.36)	0.48 (0.21-1.06)	0.75 (0.45-1.23)
**Education** ^**2**^						
Less than High School			1.66 (0.51-5.37)	3.66 (1.80-7.63)**	1.36 (0.29-4.91)	3.27 (1.56-6.90)**
High School Complete			1.07 (0.47-2.47)	1.27 (0.72-2.25)	1.07 (0.44-2.61)	1.26 (0.69-2.28)
Diploma Complete			1.40 (0.69-2.86)	1.59 (0.97-2.59)	1.30 (0.61-2.78)	1.43 (0.86-2.36)
**After tax Family Income (in $10,000)**			0.89 (0.86-0.92)***	0.90 (0.86-0.94)**	0.90 (0.84-0.96)**	0.91 (0.87-0.95)**
**Smoking** ^**3**^						
Current smoker			1.89 (1.24-2.54)**	1.43 (1.08-1.78)**	1.78 (1.21-2.36)**	1.34 (1.08-1.60)**
Former smoker			1.00 (0.55-1.46)	0.98 (0.32-1.64)	0.88 (0.41-1.36)	0.90 (0.24-1.57)
**Alcohol use** ^**4**^						
Heavy alcohol use			0.76 (0.40-1.45)	0.86 (0.56-1.32)	0.76 (0.29-1.23)	0.86 (0.54-1.18)
Moderate alcohol use			0.75 (0.35-1.65)	0.55 (0.31-1.01)	0.70 (0.23-1.17)	0.63 (0.2-1.06)
**BMI** ^**5**^						
Underweight					2.27 (0.29-17.89)	1.83 (0.42-7.95)
Overweight					1.80 (0.86-3.80)	1.45 (0.88-2.39)
Obese					2.52 (1.18-5.36)*	2.73 (1.69-4.41)***
**Engage in regular physical activity**					0.69 (0.38-1.26)	0.68 (0.45-1.01)
**Variance Partition Coefficient (VPC)** ^**6**^	8.82%***	7.31%***	6.11%**	7.19%**	5.75%**	6.08%**
**Pseudo-Akaike Information Criterion (AIC)**	19288.37	20147.68	14337.77	14769.52	13699.38	14667.89

City of Toronto, Canada 2009–2011 (n = 2411).

^‡^Model 1 estimated the bivariate association between neighbourhood income and the odds of a) MI and b) any CVDs (not shown in table).

*p < 0.05; **p < 0.01; ***p < 0.001. Bolded values are statistically significance with at least p<0.05.

^1^noise reference category = strongly disagree; ^2^Education; ^2^Education level reference category = undergrad and above; ^3^smoking reference category = non-smoker; ^4^alcohol use reference category = light/no alcohol use; ^4^noise reference category = strongly disagree; ^5^BMI reference category = normal (BMI = 18.5-24.9). ^6^This is the percentage of variance found in all neighbourhood-level variables. Our method of estimation follows the model linearization approach in Goldstein et al. [70].

Model #2 (see Table 2) reports results for outcomes 1 and 2 regressed on all neighbourhood level predictors (neighbourhood income and environmental characteristics). Neighbourhood income is still significantly associated with the 2 outcomes (p < 0.05) after adjusting for environmental characteristics. Neighbourhood level environmental factors— including access to food stores, fast food, parks and recreation (for MI only), violent crimes, living close to a major road and noise— are all significantly correlated to a higher risk of MI and other CVDs. At this point, the intercept variance component is reduced to zero, meaning that the environmental variables included here explained all the variance at the CT level. Reductions in the effect size of neighbourhood income going from model 1 to model 2 (for both outcomes) indicate that the effect of neighbourhood income is partially attenuated after adjusting for environmental variables.

Model #3 builds on model #2 by adjusting for age, visible minority status, education, after-tax family income, smoking status, and alcohol use as potential confounders. After adjustments, we found that living in CTs with the least number of food stores/supermarkets (quartile 1), compared to living in CTs with the highest numbers of food stores/supermarkets (quartile 4), is associated with an increased odds of both MI (OR = 1.23, p < 0.05) and any CVDs (OR = 1.12, p < 0.05). Living in CT with the lowest number of fast food restaurants (quartile 1), compared to living in CTs with the highest numbers (quartile 4), is associated with decreased odds of MI (OR = 0.95, p < 0.05) and any CVDs (OR = 0.97, p < 0.05). Living in CTs with the lowest amount of parks and recreational areas (quartile 1), compared to those with the highest area of parks and recreation (quartile 4), is associated with an increased odds of MI only (OR = 1.1, p < 0.05). Living in CTs with the lowest number of violent crimes (quartile 1), compared to the highest crime areas (quartile 4), is associated with reduced odds of MI (OR = 0.87, p < 0.05) and any CVDs (OR = 0.8, p < 0.001). Living in CTs with the second lowest number of violent crimes (quartile 2), compared to quartile 4, is also associated with reduced odds of any CVDs (OR = 0.88, p < 0.05). Living within 100 metres of a major road is associated with an increase odds of MI (OR = 3.86, p < 0.001) and any CVDs (OR = 2.01, p < 0.01); and participants who are disturbed by noise at home (strongly agree vs. strongly disagree) have an increased odds of MI (OR = 4.84, p < 0.01) and any CVDs (OR = 3.25, p < 0.01). The level of housing disrepair is not significantly associated with MI or any CVD outcomes. Neighbourhood income is no longer significantly associated with the odds of either outcomes with the adjustment of individual level risk factors. Out of the two health behaviour adjustment variables included in model #3 (i.e. smoking and alcohol use), only smoking significantly increased the odds of MI (OR = 1.89; p < 0.01) and CVDs (OR = 1.43, p < 0.01). The percentage of variance of the outcome found in all neighbourhood-level variables, calculated by the variance partition coefficient [70], is 6.11% for MI and 7.19% for all CVDs.

Model #4, further adjusts model #3 by adding two additional control variables: BMI and physical activity. The results of this model should be interpreted with caution because BMI and physical activity may be heavily influenced by contextual factors in our study (i.e. access to supermarkets, fast food restaurants, parks and recreational area). Thus, these variables are conceptually in the associational pathway. When these variables are added to the model, especially BMI, access to supermarkets, fast food restaurants, parks and recreational areas are no longer significantly associated with CVD. However, exposure to violent crimes, proximity to major roads, and being disturbed by noise at home remain significantly associated with MI and all CVDs (all at least at p < 0.05).

### Checking the models

Two sub-analyses based on model #4 was conducted to examine the effects of gender for two purposes: 1) to understand whether the strength of the associations between neighbourhood factors and CVD risk differed along gender lines, and 2) to validate the model by ensuring the stability of our results across gender. Since the results of the sub-group models were substantially similar to the overall model— the direction of associations and the level of significance between both gender-based sub-models and the overall model remain unchanged— the results are not shown here. These sub-model results are available upon request from the principal author.

In separate analyses, models were also specified with random slopes for gender, age, individual level income, education, and visible minority status in order to determine whether these effects vary across neighbourhoods. Allowing the slopes to vary on these covariates revealed no significant variance components suggesting that gender, age, income and educational differences are constant across the CTs. Also, no significant cross-level interactions were found.

In summary, access to supermarkets, fast food restaurants, access to parks and recreational areas (for MI only), are associated with odds of CVDs before adjusting for BMI. However, adjusting for BMI fully attenuated these associations. Rate of violent crimes, living near high traffic and noise, are associated with MI and any CVDs, even in the fully adjusted model #4. Housing disrepair was not significantly associated with CVDs.

## Discussion

The main focus of our paper is to investigate multiple neighbourhood influences guided by opportunity structure theory in an integrated approach to understand the association between the built environment and CVDs. Our study found that exposure to violent crimes, environmental noise, and proximity to a major road were independently associated with increased odds of CVDs (p < 0.05) in the fully adjusted model. Reduced access to food stores, parks/recreation, and increased access to fast food restaurants were associated with increased odds of CVDs in partially adjusted models (p < 0.05), but these associations were fully attenuated after adjusting for BMI and physical activity. Individual behavioral risk factors including smoking and obesity, treated as adjustment variables in our models, are significant risk factors for CVDs – and the mechanisms for how these factors influence CVD risk are well documented [33,71].

Our study supports the neighbourhood opportunity structure theory by showing significant associations between a range of social environmental characteristics and CVDs at the local level. Previous studies primarily focused on negative aspects of neighbourhoods, e.g. economic deprivation [27-29,72] and neighbourhood social disorganization and disorder [73-75], and limited work explored the effects of other social and built environmental factors. On the other hand, our study demonstrates the importance of measuring and modeling a broad range of environmental factors including community resources (e.g. access to healthy food and access to parks/recreational areas).

The findings in this study differ from a number of earlier studies. Our study shows that once we account for a broad range of planning-related environmental factors and individual-level covariates, the neighbourhood SES-CVD association is fully attenuated. Thus, neighbourhood SES may be acting as a proxy for more specific environmental factors. Future studies should further explore the associations between CVD and a broad set of neighbourhood characteristics including the local food environment, park/recreational space, crime rates, traffic, and noise across different settings and using a longitudinal approach to better understand the causal pathways. While the ubiquity of census and survey data may be a factor for the abundance of studies on neighbourhood SES as a factor for population health, we demonstrate here that it is relatively easy to obtain and prepare land use and planning-related spatial data for this type of study.

We had considerable debate among our team regarding the interpretation of controlling for BMI and physical activity in our study (i.e. model 4). While the majority of epidemiological investigations of CVDs include these risk factors as a matter of convention [76], we were concerned that their inclusion in our model would mitigate any neighbourhood effects that conceptually have an indirect impact on CVD through their impact on physical activity and body weight. Therefore, their inclusion may arguably be a case of over-adjustment. In the interest of full disclosure of our findings, we decided to present both the un-adjusted results (model 3) as well as the BMI-adjusted results (model 4). We stress that further research is necessary to investigate the ‘access to food, recreation, and parks’→BMI→CVD risk pathway, especially since studies on the association between food environment and obesity have decidedly mixed results [77]. The results here show that BMI may act as either a confounder or mediator for the association between ‘access to food, recreation, and parks’ and CVD risk, and further research to investigate the temporal order of events is needed to better understand the relationship among these variables.

An important limitation of this study is that the associations reported here are based upon cross-sectional data and are subject to the problems of 1) not being able to discern the direction of causation, 2) potential unadjusted individual level confounders, and 3) ignoring participants’ earlier life influences that may have impacted the risk of adverse CVD outcomes. Due to health-selected migration, residents may choose the neighbourhoods that they live in based on their health-related characteristics [73]. Another limitation to our study is that our sample is missing individuals who do not survive CVD events or those who are cognitively impaired by CVD events to the extent that they cannot participate in a 1.5 hour face-to-face interview. It is likely that these individuals are not randomly distributed but rather, are over represented among those with low socioeconomic position. In this case, our estimates for socioeconomic differences may be conservative. On the other hand, it is also possible that low medical literacy or poor recall may be over represented among those who have lower socioeconomic position. In this case, our estimates for socioeconomic position would be biased away from the null. Residential noise was found to be important in our study for CVD risk. Future research should examine the whether there are significant differences between noise that originates in the home versus noise from the local neighbourhood. In our study, CVD risk was lower in low versus high crime neighbourhoods. Our data did not enable us to examine the mechanism responsible for the association for crime and CVD risk. Future studies might determine whether risk varies by more specific crime types in the neighbourhood.

## Conclusions

Neighbourhood opportunity structure theory provides a useful framework to investigate the local level environmental correlates of population health. Our study gives evidence to support the theory by showing significant associations between a range of environmental characteristics and CVD risk, which sheds light on the potential pathways that should be tested in future longitudinal studies. This information is also useful for informing future interdisciplinary research across the fields of urban planning and public health, since this study provides evidence to show how land use decisions may impact population health. Public health research often focuses on interventions to modify health behaviors, and social and built environmental factors are less commonly considered. The findings suggest that the reintegration of urban planning and public health may help to inform prevention for ‘sick populations’ [78], since many of the environmental determinants examined in our study are shaped by land-use decisions [79].
